# The TDGL Module: A Fast Multi-Scale Vision Sensor Based on a Transformation Dilated Grouped Layer

**DOI:** 10.3390/s25113339

**Published:** 2025-05-26

**Authors:** Leilei Xie, Fenghua Zhu, Zhixue Wang

**Affiliations:** 1 School of Rail Transportation, Shandong Jiaotong University, Jinan 250357, China; 13093322015@163.com; 2 Institute of Automation, Chinese Academy of Sciences, Beijing 100190, China; fenghua.zhu@ia.ac.cn

**Keywords:** spatial pyramid pooling, data normalization, object detection, expansion rate

## Abstract

Effectively capturing multi-scale object features is crucial for vision sensors used in road object detection tasks. Traditional spatial pyramid pooling methods fuse multi-scale feature information but lack adaptability in dynamically adjusting convolution operations based on their actual needs. This limitation prevents them from fully utilizing spatial hierarchies and contextual information. To address this challenge, we propose a Transformation Dilated Grouped Layer (TDGL) module, a fast multi-scale vision sensor based on deep learning, designed to enhance both efficiency and accuracy in road target feature extraction networks. The TDGL is built upon the Global Layer Normalization Convolution (GLConv) unit, which mitigates internal covariate shift by introducing scaling and offset parameters, modifying dilation strategies, and employing grouped convolution. These improvements enable the network to distinguish features at different scales effectively while optimizing spatial information processing and reducing computational costs. To validate its effectiveness, we integrate the TDGL module into the backbone of several YOLO models, forming the TDGL Net feature extractor. The experimental results obtained on the BDD100K dataset show that the mAP of the TDGL net reaches 40.3% with around 3.1M parameters. The inference speed of the TDGL net after transformation optimization reaches 58 FPS, which meets the requirement for the real-time detection of road obstacle targets by autonomous vehicles.

## 1. Introduction

**Figure 1:**
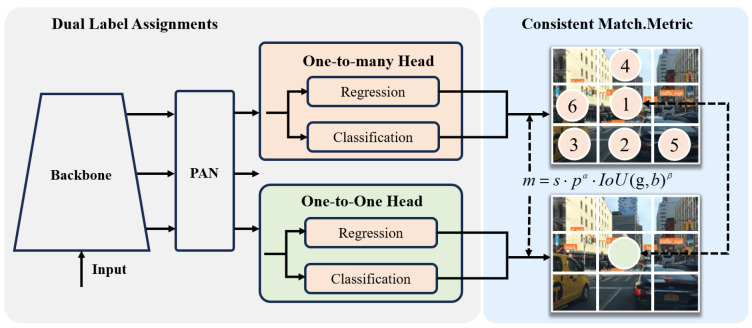
Illustration of road object detection framework.

Multi-scale vision sensors, as a fundamental and critical area of research, drive technological innovation in several fields such as smart cities, autonomous driving, and intelligent surveillance. With the continuous development of convolutional neural networks CNNs [1], the performance of target detection based on vision sensors has achieved significant improvement, but their computational burden has also increased significantly and cannot satisfy the real-time requirements of autonomous driving. The candidate region-based two-stage convolutional neural network R-CNN [2], Fast R-CNN [3], and Faster RCNN [4] can be regarded as high-performance versions of a CNN and have achieved higher detection accuracy on datasets such as KITTI [5], VOC 2012 [6], and MS COCO [7]. However, the main challenge that they are faced with is the strict limitations on their input image size. Spatial pyramid pooling has been proposed to solve this problem, and a combination of classifiers can effectively detect and localize target objects. To improve the accuracy of the detector, a deeper layer is usually adopted to achieve better results. Ma et al. [8] introduced a pyramid cavity convolution module in parallel with the RESA module to enhance the model’s receptor field to enrich and extract global spatial feature information, but the detection speed was decreased to a low level by this. S. alaiarasan et al. [9] used a deep convolution neural network containing 12 nested processing layers for object detection. Wen et al. [10] proposed an MSADark module with global attention and the location attention-weighted feature fusion network LAFFN to enhance network feature representation for target perception in automatic driving. The main deficiency of the module lies in the huge amount of computation required. Niu et al. [11] proposed a lightweight method based on the improved YOLOv8 to significantly improve the detection performance of a model through multidimensional optimization. The method introduces an efficient multi-scale attention mechanism while combining it with the SPD-Conv module to alleviate the problem of the loss of fine-grained information about targets. However, in real-time detection scenarios, this model still needs to be optimized to enable multi-target feature extraction in dynamic scenes. Figure 1 illustrates road object detection using a dual label assignment strategy, which balances precision and computation. Liu et al. [12] carried out a detailed analysis of the batch normalization layer and integrated low precision, range batch normalization, and block floating point technology to effectively reduce the running time overhead in the batch normalization process. All these methods are based on increasing the depth of convolution and greatly increase the computational burden of the model and reduce its inference speed.

To achieve the optimal balance between accuracy and real-time performance, this paper tries to study the operation mechanism of deep neural networks and focuses on the improvement and optimization of basic convolutional units, considering that the fine-tuning of parameters in any layer of the convolutional neural network may cause a significant shift in data distribution in subsequent layers. After multiple parameter updates and training transfers, the changes in input distribution in the rear layer will intensify [13]. Variable data distribution will not only cause the model to behave unstably in the training process, affecting the quality and speed of the model convergence, but it will also affect the scale and variance of the data and tends to cause the gradient to vanish or explode in multilayer networks, especially when saturated activation functions, such as Sigmoid or Tanh, are used. Therefore, effective strategies should be adopted to stabilize the data’s distribution and optimize the network structure to improve the performance and generalization ability of the model.

This paper attempts to make a lightweight multi-scale vision sensor feature extraction network with better performance by using an optimized GLConv convolutional module as its basic unit and multi-branch architecture design to obtain multiple receptor fields. In the network, the TDGL module replaces the spatial pyramid module of YOLO [14] to build the TDGL Net feature extractant, which is highly portable and can be used in multiple target detection models. The main contributions of this paper are as follows:

- A new standard convolutional unit, GLConv, is designed, and non-zero values are added to the batch variance to enhance the stability of feature information extraction.
- A multi-branch TDGL detection module based on normalized convolutional units is proposed. The TDGL adopts an integrated and more flexible convolutional kernel mechanism, which improves its performance in extracting feature information from targets of multiple sizes with limited computational resources and enhances the detection capability of visual sensors for multi-scale targets on roads.
- Experiments are carried out on the BDD100K benchmark, and the effectiveness of our network is verified.

The rest of the main sections of this article are arranged as follows: Related work is shown in Section 2. Section 3 elaborates on the TDGL network used in multi-scale vision sensors and provides an in-depth analysis of the proposed strategy. Comparison experiments, ablation experiments, and relevant analyses are described in Section 4. Finally, the conclusion is presented in Section 5.

## 2. Related Work

Capturing multi-scale target features effectively is essential to improving the performance of vision sensors. To improve their feature extraction ability, the spatial pyramid pooling (SPP) proposed by He et al. [15] fuses features of different levels through multi-scale pooling operations, significantly improving the model’s adaptability to scale changes. However, the parallel pooling structure of SPP has high computational complexity and struggles to meet the real-time requirements. Therefore, Liu et al. [16] introduced a simplified version of SPP (SPPF) into their model. SPPF enhances the original spatial pyramid pooling by transforming the computationally intensive parallel pooling layer into a more efficient serial structure, improving accuracy while increasing processing speed. SPPF employs a progressive pooling strategy that begins by pooling a large area before gradually reducing the pooling window size. This approach minimizes redundant operations while effectively capturing features at various scales. Inevitably, our ability to adjust the receptive field is still limited by the fixed pool window. Max pooling is calculated as follows:

(1) $$z_{i}=\mathrm{MaxPool}2d\left(z_{i-1},k=5,s=1,p=2\right)$$

where $z_{i}$ is the result of the i time pooling operation; $z_{0}$ = *Y*, where *Y* is the initial convolution dimension reduction value of the feature graph; and p is the filling value, that is, the zero layers added to the edge of the input feature graph to maintain the size of the output feature graph.

We apply adaptive maximum pooling followed by adaptive average pooling to the output feature map in succession, as detailed below:

(2) $$\left\{\begin{array}{c} a_{\max}=\mathrm{AdaptiveMaxPool}2d\left(z_{3},o=1\right)\\ a_{\mathrm{avg}}=\mathrm{AdaptiveAvPool}2d\left(a_{\max},o=1\right) \end{array}\right.$$

where $a_{\max}$ and $a_{\mathrm{avg}}$ are the processed results, *O* is the output size, and *O* = 1 indicates that adaptive pooling will convert input feature maps of any size into 1 × 1 feature maps.

The results of all pooling operations are spliced along the channel dimension, and the spliced feature diagram *f* is obtained:

(3) $$f=\mathrm{Concat}(y,z_{1},z_{2},z_{3},a_{\max},a_{\arg})$$

We convolve *f* and apply the activation function to obtain the final output feature graph:

(4) $$\left\{\begin{array}{c} Z\left[h,w,c\right]=\sum_{c^{\prime }}^{C_{\mathrm{in}}}W^{\left(c\right)}\left[0,0,c^{\prime }\right]\times f\left[h,w,c^{\prime }\right]+B\left[c\right]\\ Y[h,w,c]=\mathrm{SiLU}(Z[h,w,c])\\ C_{\mathrm{in}}=c^{\prime }\times 6+c^{\prime }/2 \end{array}\right.$$

where Z[h,w,c] is the result after the convolution operation, Y[h,w,c] is the value of the output feature map, *c* is the output channel number, $C_{\mathrm{in}}$ is the number of channels in the feature map, $W^{\left(c\right)}\left[0,0,c^{\prime }\right]$ is the cth output channel weight matrix element corresponding to the position of the input channel $c^{\prime }$, $f[h,w,c^{\prime }]$ is the value of the feature map at the corresponding position, and B[c] is the bias term of output channel *c*.

Some researchers have tried to optimize multi-scale feature fusion through dynamic convolution strategies. For example, Chen et al. [17] proposed adaptive spatial pyramid pooling (ASPP), which uses void convolution to flexibly adjust receptive fields, but its fixed setting of the void rate limits its adaptability to complex scenes. Similarly, the field enhancement network (RFB-Net) designed by Li et al. [12] has an improved ability to detect small targets through multi-branch cavity convolution, but the balance between computational efficiency and accuracy is not solved.

In the process of feature fusion in multi-scale vision sensors, with the deepening of feature information transmission in the network, the distribution of the feature graphs after fusion will become more complex due to the influence of multiple factors such as the update of the network parameters and the action of an activation function and pooling operation, which may aggravate the shift of internal covariables. Huang et al. [18] proposed a method for measuring the displacement of internal covariates using the EM distance, derived the upper and lower bounds of the method, and combined the output with adjustable parameters to further constrain the data’s distribution and reduce information loss. This approach brings the performance of the detection model close to that of the two-stage scheme, sometimes even exceeding it, but it has a great impact on the processing speed.

To solve these problems, this paper reconstructs the multi-scale feature convolution module and designs a new normalized convolution unit. By introducing normalized parameters and a targeted expansion strategy, multi-scale feature fusion is realized efficiently and spatial information processing is optimized to reduce the computational overhead.

## 3. Methods

**Figure 2:**
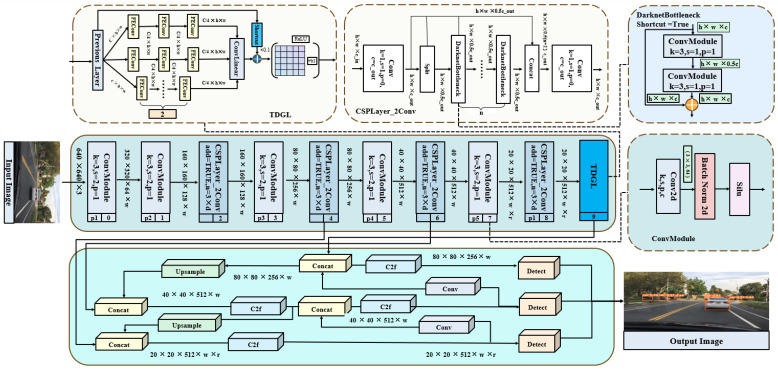
Improved one-stage network architecture.

The road detection task for vision sensors involves multiple detection targets, which places a large computational burden on edge devices. This study aims to enhance the performance of single-stage detection models without increasing the computational load. To improve training efficiency and accelerate convergence, we avoid simply increasing the convolution depth of the backbone network. Instead, we focus on optimizing the convolution units, implementing batch normalization, adjusting the weight of historical data when updating the running mean and variance, and incorporating learning for affine transformation parameters during normalization. The TDGL feature extraction module utilizes a multi-branch convolutional network structure with GLConv as its foundational unit. Each branch employs different spatial expansion rates to capture features at multiple scales. Below, we provide specific parameter information and testing solutions. This structure is inspired by the Receptive Fields Block, which allows for the efficient extraction of structural and texture information from multi-scale objects in images by adjusting the receptive field of the detection module. As a result, it demonstrates a better performance than traditional spatial pyramid networks. The TDGL module is integrated at the end of the YOLO v8 backbone network, creating an efficient TDGL Net feature extractor, as illustrated in Figure 2. In the figure, a sketch of the structure of the TDGL module is presented in the light yellow box on the left-hand side, while the structure of the CSPLayer in the backbone network is shown in the light yellow box on the right-hand side. The blue part in the top right corner contains the structural diagram of DarknetBottleneck, the dark green box in the middle contains the backbone structure of the network, and the bright green box at the bottom displays the neck and detection header parts of the network.

### 3.1. TDGL Feature Extraction Module

The TDGL module significantly enhances the feature extraction capabilities of convolutional neural networks and consists of a multi-branch convolutional block. Its structure comprises two main components: a multi-branch convolutional layer with various kernel sizes and an extended pooled convolution layer, which is added at the end. This design effectively implements a multi-receptive field structure using multiple kernel sizes, outperforming fixed-size shared convolution kernels.

The specific design of the TDGL module is inspired by Inception-ResNet V2 [19]. First, to reduce the number of channels in the feature map, a bottleneck structure with 1 × 1 GLConv convolutional layers is employed in each branch. Second, two stacked 3 × 3 convolutional layers replace larger convolution kernels, minimizing the depth of nonlinear layers and reducing computational complexity. Finally, a 1 × n followed by an n × 1 convolutional layer is utilized instead of the traditional n × n convolutional layer, employing a shortcut spanning approach at the end. The branch convolution operation assumes that the input is a *C* channel, and then each pixel in the $C^{\prime }$ channel output map at (y,x) is evaluated as follows:

(5) $$O_{y,x}=\sum_{i=-k_{h}^{\prime }}^{k_{h}^{\prime }}\sum_{j=-k_{w}^{\prime }}^{k_{w}^{\prime }}W_{k_{h}^{\prime }+i,k_{w}+j}\cdot I_{y+i,x+j}$$

where *x* and *y* represent the x and y axes of the output map; $k_{h}$ and $k_{W}$ are the size of the nucleus, which represent thes convolution filter; $I_{v+i,x+j}\in R^{C}$ and $O_{y,x}\in R^{C^{\prime }}$ are the input and output, respectively. The bias term of the convolution is ignored in this equation for simplicity of presentation. The primary goal of employing a convolutional layer structure is to generate higher-resolution feature maps that capture a broader range of contextual information while maintaining a constant number of parameters. This design is also evident in the single-stage detector SSD, which enhances its detection speed effectively.

**Figure 3:**
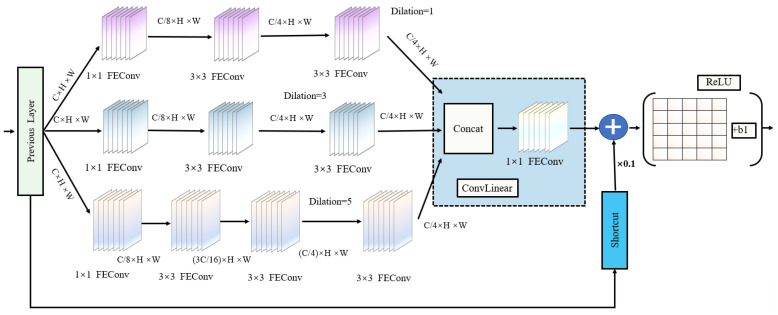
Network structure of TDGL feature extraction module.

The detailed structure of the TDGL module is illustrated in Figure 3. Following the research of Liu et al. [12], extended convolution is employed to simulate the effects of receptive field deviation in the human visual cortex. This design enables the module to capture a diverse range of features across various spatial levels, thereby enhancing the detection of different sizes of objects across contextual scenes. In each branch, convolutional layers with specific kernel sizes are combined with corresponding expansion layers, and the outputs of these branches are fused by summing to create a richer feature representation. The three branches extract features at different scales using different sizes of convolution kernels or different expansion rates. Finally, the output feature maps of multiple branches are merged along the channel dimensions through the operation of Concat (channel splicing), as shown in Equation (4). Compared with element-by-element addition, Concat does not destroy the feature distributions of each branch and directly retains all the output information to avoid information loss. The kernel size expansion rate exhibits a positive correlation with both size and the eccentricity of the visual cortex’s receptive field. Adjusting the expansion rate effectively enlarges the kernel size from k×k to $k_{\varepsilon }$ without increasing the number of parameters or the computational load. The expression is as follows:

(6) $$k_{\varepsilon }=k+\left(k-1\right)\left(r-1\right)$$

where $k_{\varepsilon }$ represents the size of the equivalent expansion convolution kernel and *r* represents the size of the expansion rate. The formula for calculating the receptive field of the current layer is as follows:

(7) $$RF_{i}=RF_{i-1}+\left(k-1\right)\times \prod_{i=1}^{i}Stride$$

where $RF_{i}$ is the receptive field of the current layer, $RF_{i-1}$ is the receptive field of the previous layer, and $\prod_{i=1}^{i}Stride$ is the product of the steps of all previous layers.

Finally, each module merges the original input with the output from feature fusion through a residual connection, forming a spatial convolution array that enhances network information transfer and training convergence. The TDGL module consists of three parallel branches. In this paper, the default step value for controlling the convolution operation is set to 1, and the reduction factor for the number of channels in the middle layer is set to 8 to minimize parameters. The scaling factor for shortcut connections is also set to 0.1. Each branch has a different convolution expansion rate: the last layer of Branch 1 uses a 3 × 3 convolution with an expansion rate of 1, focusing on smaller image regions to preserve detailed features. Branch 2’s final convolutional layer is set to an expansion rate of 3, allowing it to capture medium-scale features such as partial targets or backgrounds. Meanwhile, the last layer of Branch 3 has an expansion rate of 5, enabling it to capture large-scale global information and better understand the overall structure and background of the image.

The TDGL module is designed to capture multi-scale contextual information by combining more flexible and efficient mechanisms. It allows us to avoid replacing the SPPF module with a deeper or denser network that incurs significant computational costs. This paper proposes a deep learning-based single-level framework for a visual sensor feature extraction network and integrates the TDGL module to enhance the lightweight feature extraction backbone. This approach maintains fast detection while improving accuracy. The TDGL module boasts high compatibility and has been successfully applied to multiple versions of YOLO, with the primary modification being the replacement of the spatial pyramid module at the end of the backbone with TDGL.

The SPPF module is situated in layer 9 of the backbone network. Its input is typically processed through three max pooling layers with a kernel size of 5, following a 1 × 1 convolution. In this paper, we remove the max pooling layer and restrict the convolution kernel size to less than 3 to reduce the computational burden. Max pooling decreases the size of the feature map by selecting the maximum value from each pooling window, which can result in a loss of detailed information. Additionally, gradient propagation is limited to the maximum position within the pooling window, leading to an uneven gradient flow and potentially hindering model training. By carefully selecting the step size and filling strategy, we can retain more information from the input feature map. This approach allows for more uniform gradient propagation back to the earlier layers of the network, facilitating efficient parameter tuning.

### 3.2. GL Feature Extraction Convolution

**Figure 4:**
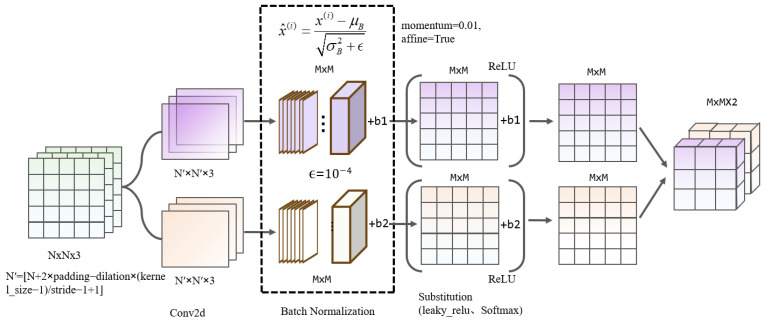
GLConv structure in terms of normalized convolutional units.

Changes in the distribution of network layer activation inputs can slow learning and destabilize the model’s performance during training. The GLConv module is designed to enhance the speed and accuracy of feature extraction in convolutional neural networks while minimizing the impact of internal covariate shift on training stability. Its structure is illustrated in Figure 4.

We improve the feature output after the two-dimensional convolution layer by incorporating an integrated batch normalization layer. A small non-zero value is added to the batch variance to enhance numerical stability, and the parameters are adjusted accordingly. Batch normalization standardizes the features of each instance using the mean and variance statistics of the batch data, ensuring that the mean is 0 and the variance is 1. This keeps the inputs to each layer relatively stable and reduces internal covariate shift. The batch data dependence of this method enhances network convergence, thereby accelerating the speed of convergence and improving the model’s generalization ability. Input image feature values $x=(x^{\left(1\right)},x^{\left(2\right)},\ldots ,x^{\left(m\right)})$ are normalized using the following steps:

(8) $${\hat{x}}^{\left(i\right)}=\frac{x^{\left(i\right)}-\mu_{B}}{\sqrt{\sigma_{B}^{2}+\epsilon }}$$

(9) $$\mu_{B}=\frac{1}{m}\sum_{i=1}^{m}x^{\left(i\right)},\;\sigma_{B}^{2}=\frac{1}{m}\sum_{i=1}^{m}{\left(x^{\left(i\right)}-\mu_{B}\right)}^{2}$$

where m represents the number of samples in a batch, x is the input feature, i represents the sample i, $\mu_{B}$ is the mean of the data features of the training batch, $\sigma_{B}^{2}$ is then the variance of the data features, ϵ is a small positive value that prevents the denominator from being zero when calculating normalization, and ${\hat{x}}^{\left(i\right)}$ is the result of the normalization.

The value ϵ usually defaults to $10^{-5}$. Consider that BDD100K is a dataset containing various target classes with complex features. Conditions such as bad weather or uneven lighting in the images may result in high or low variance in the input data. In this paper, the value of ϵ is adjusted to $10^{-4}$. Increasing the value of ϵ improves numerical stability and helps to deal with the differences in image data captured under different environmental conditions. To minimize the impact of noise on the normalization process, we made slight adjustments to the momentum values used for computing the sliding average. While normalized data enhance training stability by maintaining zero mean and no unit variance, these properties may not always align with the feature expression needs of the current layer. Therefore, this paper sets the affine Boolean parameter to true and introduces scaling and offset parameters to perform an affine transformation on the normalized data, allowing for the recovery or retention of certain characteristics from the original data. The transformation formula is as follows:

(10) $$y_{i}=\gamma {\hat{x}}_{i}+\beta$$

where γ is a scaling parameter that directly affects the scale of the gradient, β is an offset parameter that regulates the bias of the gradient, and $y_{i}$ represents the final output feature.

After the convolution operation, the ReLU activation function is applied to the output feature maps of each convolutional layer and the ReLU forward propagation formula is as follows:

(11) $$x_{j}^{l}=f\left(\sum_{i\in M_{j}}x_{i}^{l-1}*w_{ij}^{l}+b_{j}^{l}\right)$$

(12) $$y_{k}\left(i,j\right)=\left\{\begin{array}{c} x_{k}^{l}\left(i,j\right),x_{k}^{l}\left(i,j\right)>0\\ 0,x_{k}^{l}\left(i,j\right)\leq 0 \end{array}\right.$$

where $x_{j}^{l}$ represents the jth feature graph in the l layer, $f\left(x\right)$ is the nonlinear activation function, $M_{j}$ is the set of input images, ∗ represents the convolution operation, $w_{ij}^{l}$ is the weight matrix of the convolution kernel, $b_{j}^{l}$ is the bias value, and $y_{k}\left(i,j\right)$ is the output.

The backward propagation of ReLU requires it to be one of the layers of the network. Taking $x^{l}$ as the output of layer *l*, the partial derivative $\delta^{L}$ of the loss function *L* related to the output of the *l* layer is formulated as follows:

(13) δL=∂L∂xl=δl+1.∂ReLU(xl)∂xl=δl+11,if xl>00,if xl≤0

One major drawback of ReLU is that the gradient is zero when the input is negative. This leads to neuron death [20], which prevents these neurons from being updated during the training process. To solve this problem, Leaky ReLU [21] was introduced into the convolutional unit. Leaky ReLU allows a small negative slope when the input *x* is negative, thus alleviating the problem of neuron death, and its forward and backward propagation formulas are as follows:

(14) $$Leaky\left(x\right)=\left\{\begin{array}{c} x,x>0\\ leak^{*}x,x\leq 0 \end{array}\right.$$

(15) $$\delta^{L}=\delta^{l+1}.\frac{\partial Leaky(x^{l})}{\partial x^{l}}=\delta^{l+1}\left\{\begin{array}{c} 1,if\;x^{l}>0\\ leak,if\;x^{l}\leq 0 \end{array}\right.$$

where ∗ is the multiplication sign and leaky is a small positive constant, usually around 0.01 [22].

### 3.3. Training Strategies

The TDGL multi-scale vision sensor was developed using the PyTorch framework and integrates various components from the YOLO multi-version open-source repository. This paper’s training strategy primarily follows that of the baseline model, which includes Mosaic data augmentation, anchor box selection and matching strategies, multi-scale prediction, and a specific loss function. The TDGL network’s loss function comprises three main components: border loss, inter-class loss, and target loss, with the CIoU loss function specifically used for the border loss. This loss function takes into account the centroid distance, overlap ratio, and aspect ratio of the detection frame and is defined as follows:

(16) $$CIoU=IoU-\left(\frac{\rho^{2}\left(b,b^{gt}\right)}{c^{2}}+\alpha v\right)$$

(17) $$\alpha =\frac{v}{(1-IoU)+v}$$

(18) $$v=\frac{4}{\pi^{2}}{\left(\arctan\frac{w^{gt}}{h^{gt}}-\arctan\frac{w}{h}\right)}^{2}$$

where b is the center point of the predicted box, c is the diagonal length of the smallest closed box covering the predicted box and the true box, $b^{gt}$ is the center point of the true box, α is the trade-off coefficient, v is used to measure the consistency of the aspect ratio, and ρ is the Euclidean distance between the center point of the true box and the center point of the predicted box. w, h, $w^{gt}$, and $h^{gt}$ in the relevant formula denote the length and width of the predicted box and the true box, respectively. The AdamW optimizer is employed during training to adjust the model weights and minimize the loss function. The learning rate is set to 0.01 for v8 and v5 and 0.001 for v3, enhancing the effectiveness of the TDGL module’s embedding. Only the v8 model utilizes the official weights; the others do not use initial weights. More detailed experimental parameters are provided in the Experiments section.

## 4. Experiments

**Figure 5:**
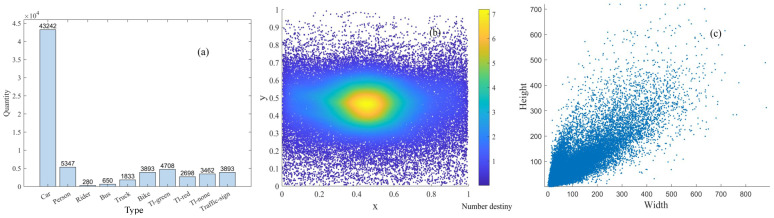
Visualization of BDD100K dataset. (**a**) Number of targets in each category. (**b**) Distribution diagram of label frame center points. (**c**) Distribution diagram of label frame sizes.

To comprehensively evaluate the improvement in performance created by the proposed TDGL feature extraction module when used in YOLO series object detection models, this section tests these models on the BDD100K [5] dataset. In this experiment, we randomly selected 4220 images as the training set and 600 images as the validation set. Various original algorithm models and their improved versions were compared. Before the experiment, a small number of training classes and two categories of non-signal traffic light targets were removed to balance the dataset. A traffic scene dataset was constructed, featuring 10 target types: person, rider, car, bus, truck, bike, tl_green, tl_red, tl_none, and traffic_sign. The visualized data information from the BDD100K dataset is presented in Figure 5. Here, “quantity” represents the number of categories in the dataset, while the x and y axes depict the distribution of the labeled box positions. The label box size distribution graph in Figure 5c clearly shows that target sizes are primarily concentrated around 200 pixels, indicating a high prevalence of small targets within the dataset.

### 4.1. Training Settings

The experimental environment used in this article is the Pytorch 1.10.1 deep learning framework and the Python 3.8 programming language. The graphics card used in the hardware section is the NVIDIA GeForce GTX 1660 Ti. The CPU model is an Intel(R) Core(TM) i7-9700, and the system storage size is 16.0 GB. The detailed hyperparameter settings used for training are shown in Table 1, and the rest of the parameters are consistent with the official model.

The models’ detection performance is evaluated using the mean average precision (mAP), which provides a comprehensive assessment of the algorithms by calculating their mean accuracy at various confidence thresholds. The primary evaluation metrics used include the recall rate, mAP@0.5, and mAP@0.5:0.95. The key difference between these mAP metrics lies in their IoU thresholds; mAP@0.5 considers only an IoU threshold of 0.5, while mAP@0.5:0.95 averages the mAP across IoU thresholds from 0.5 to 0.95. Additionally, speed of performance is measured using frames per second (FPS), indicating the number of images the model can detect per second. The mAP calculation formula is as follows:

(19) $$mAP=\frac{1}{c}\sum_{i=1}^{c}AP_{i}$$

where C is the number of categories and $AP_{i}$ denotes the accuracy rate of the ith category, which is calculated as follows:

(20) $$AP_{i}=\int_{0}^{1}\left(P_{i}\cdot R_{i}\right)$$

where $P_{i}$ and $R_{i}$ denote the accuracy and recall of detection category i. The methods used to calculate precision and recall are shown in Formulas (9) and (10) respectively:

(21) $$Pression=\frac{TP}{TP+FP}$$

(22) $$Recall=\frac{TP}{TP+FN}$$

where FP denotes the number of negative samples that were incorrectly identified as positive samples, TP denotes the number of positive samples that were correctly identified, FN denotes the number of positive samples that were incorrectly identified as negative samples, and the ratio of the number of positive samples to the total number of samples is calculated.

### 4.2. Experiments into Replacing Backbone

The TDGL module was integrated into a series of YOLO releases. Specifically, in v5, TDGL took the place of a spatial pyramid structure at layer 10 at the end of the backbone network, with the same backbone network depth as in Figure 2. The location of the TDGL’s embedding in v6 is in the backbone network ERBlock5, one layer below the RepVGG Block and RepBlock. Due to hardware limitations, TDGL was configured in YOLOv3-tiny for testing. The standard v3 structure was not evaluated, as its replacement location differs from the others, being situated in layers 8 and 10 of the backbone. Additionally, replacements were made in layer 1 of the HEAD section by removing the original convolution layer. The weight file chosen for the experiments is based on n, which offers a balanced trade-off between size and detection performance.

Table 2 shows the detection results when replacing the GLConv in TDGL with the standard Conv + BN in v6 and v8, as well as the test results after replacing the TDGL module with the YOLO series. For a fair comparison, this paper maintains consistency in the training parameters used for the model before and after the improvement, and all experiments are conducted in the same environment. During the experimental process of integrating the TDGL module into multiple versions of YOLOv3, v5, and v6, this paper uses the same training strategy, including consistent data enhancement methods, optimizer selection, and learning rate scheduling. The original models and improved models used the same training strategy and hyperparameter settings during the training process.

The data indicate that the improved models achieved a higher detection accuracy, with only a slight increase in the number of parameters and floating-point computations. The module constructed using the standard Conv + BN is similar to the original model in terms of its number of parameters, but its detection results are slightly lower than those of the original model. Improvements in v3 have made the number of parameters and calculations smaller, improving its weight. By integrating the TDGL layer, the improved model YOLOv8-TDGL outperforms other models with a 40.3% mAP while maintaining a real-time speed of 58 frames per second. Even its accuracy is higher than that of the advanced model Faster-RCNN [4] in the two-stage framework. The feature information extraction capability of TDGL is superior to the SPPF module using a maximum pooling layer. Its performance is superior compared to that of the v6 model using the deepened base backbone network approach, which also retains an excellent processing speed.

**Figure 6:**
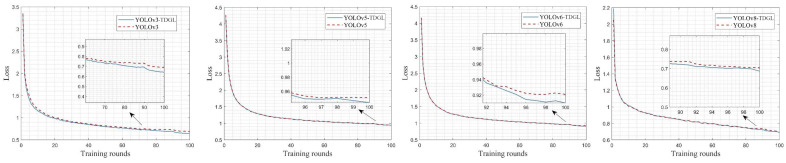
Loss function value change curve.

The improved model was trained alongside the original v3, v5, v6, and v8 models for 100 epochs on the BDD100K dataset. The change curve of the loss function is depicted in Figure 6, illustrating the model’s convergence during training. Notably, all loss values in the improved models show a reduction compared to their original models, indicating that the TDGL Net feature extractor enhances the networks’ ability to learn more complex and abstract features. This reduction in loss helps decrease the deviation between the predicted and actual bounding boxes, thereby improving the model’s generalization capability. Specifically, the loss function value for YOLOv8-TDGL, which achieved the best detection performance, dropped to 0.68. The loss function value for the v6 model also significantly decreased after the improvements, likely due to the original model’s inability to fully learn the features of the image targets during training.

**Figure 7:**
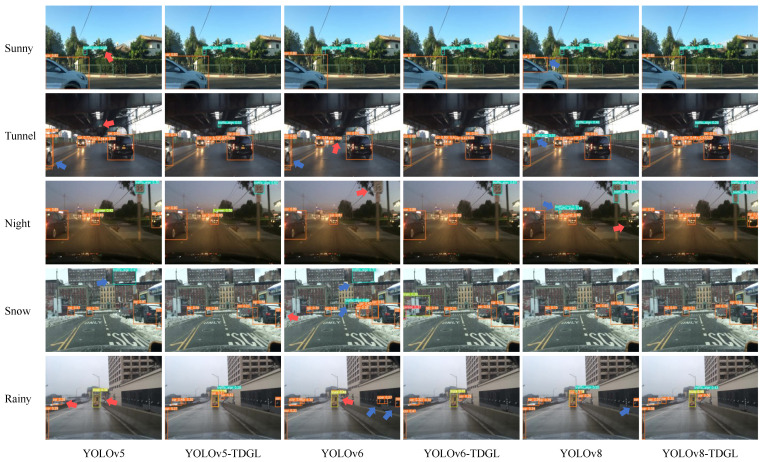
Comparison of multi-scale detection results.

Figure 7 shows the detection results for five representative scenes (Sunny, Tunnel, Night, Snow, and Rainy) before and after model optimization. Blue arrows indicate misdetected objects, while red arrows mark missed detections. The original v5 model exhibits a higher incidence of missed detections for small targets, such as traffic signs and distant vehicles, and it mistakenly identifies buildings as traffic signs. The same issues are observed in the v6 model. In contrast, the improved models effectively address these problems, enhancing detection accuracy. Notably, YOLOv8-TDGL demonstrates the most precise localization of target edges and overall detection performance. The integration of the TDGL Net feature extractor allows the model to more accurately locate targets, resulting in fewer false positives and missed detections. Additionally, it provides a higher confidence level for detected targets, significantly improving the models’ overall detection performance.

**Figure 8:**
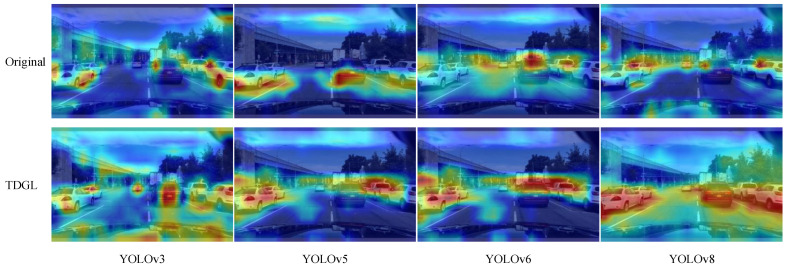
Comparison of model heat map effects.

Multiple sets of weights were used to generate heat maps for the same road image before and after enhancement to illustrate the TDGL module’s improvement of the vision sensor’s feature extraction efficiency. The results are presented in Figure 8. The intensity of the red color in the heat map indicates the level of attention the model pays to specific regions, with darker red signifying greater computational weight and a more significant impact on detection outcomes. In the original v3 plot, the red areas are small and light, indicating that the model only sporadically focuses on a limited portion of the target, while neglecting the center of the image. After integrating TDGL, the red region covering the target is larger, demonstrating that the model more accurately concentrates on the feature information of road targets. These results confirm that the TDGL module enhances the extraction and learning of target feature information, effectively improving the model’s detection performance.

### 4.3. Experiments Comparing Our Approach to SOTA Methods

To evaluate the performance of the improved model, this paper experimentally compares the YOLOv8 model with the embedded TDGL module against various mainstream target detection models on the BDD100K dataset. The results are presented in Table 3. YOLOv8-TDGL demonstrates an outstanding detection performance across multiple indicators. Compared to the SSD, which also employs a single-stage strategy, YOLOv8-TDGL shows significant improvements in various metrics. Notably, its mAP@0.5 surpasses that of the two-stage method Fast R-CNN, which utilizes a deeper network, and it significantly outperforms other methods in terms of precision.

Overall, the detection frame rate of the single-stage YOLOv8 model reaches 58 FPS, far exceeding that of the two-stage detection models. Although RetinaNet, which uses ResNet-18 as its base network, exhibits faster inference speeds due to it having fewer convolutional layers, YOLOv8-TDGL maintains a competitive edge. The recall metrics for v3 and v5 are lower than those of the two-stage approach, likely due to the fixed number of anchor boxes used for target prediction. This design and matching strategy may lack flexibility, resulting in poor alignment between real targets and predefined anchor boxes, especially when dealing with objects of varying scales and shapes. Consequently, this leads to an increased number of missed detections.

### 4.4. Ablation Experiments

This section includes a series of ablation experiments. This study investigated the effects of the expansion rate, module depth, channel reduction factor, and activation function on the detection performance of the normalized convolution units employed in the TDGL module. Experiments were conducted on a v8 network, with results presented using various evaluation metrics. Table 4 displays the experimental results for different convolutional kernel sizes. A total of 15 groups of expansion rates were compared to assess how varying sizes influence the feature extraction capabilities of the normalized convolution units. Notably, these experimental improvements do not alter the model’s parameter count. Three of them, 1\2\6, 1\2\5, and 1\3\6, produced unsatisfactory performance test indicators and their results are not shown in the table. The data in the table show that the Leaky ReLU activation function produces the best detection results when the expansion rate is set to 1, 3, or 5, the three-branch depth is 3, 3, and 4, and the channel reduction factor is 4.

On a sunny day, the details of the near targets are clear, and the small receptive field, with an expansion rate of 1, can accurately capture these details, such as the text on a license plate and the pattern of a traffic sign. Distant vehicle targets occupy a small area in the image, and the large receptive field of expansion rate 5 captures the vehicle’s overall shape and positional information. The expansion rate of 3 in the medium receptive field strikes a balance between local details and global information, which helps to differentiate between the target and the background and reduces background interference, such as the misdetection of buildings. On the other hand, the light inside the tunnel is dim, and the contour of near vehicles may not be clear enough for detection. A small receptive field can focus on nearby areas and enhance the detection of the vehicles’ contours. At the tunnel’s exit, the light varies greatly, and a large receptive field can capture a wider range of information about the light variations, which helps the model adapt to such variations and maintains its stable detection performance. In the night scene, headlights and pedestrian edges generate noise, and a small receptive field can locate these areas more accurately and suppress the effect of that noise through appropriate processing. At night, the overall light is weak, but large obstacles may create obvious light intensity distribution regions in the image, meaning the large receptive field can capture information about these regions to realize the localization of large obstacles.

Simply increasing the receptive field by raising the expansion rate does not enhance model performance. While a larger expansion size extracts a broader range of features, it also introduces excessive redundant pixel information from the background. This can cause the response region to spread across the entire feature map, impairing the model’s ability to accurately localize targets and increasing its overall computational demands. The Leaky ReLU activation function helps maintain a smooth gradient interval, facilitating easier backpropagation and gradient updates, which ultimately improves the model’s performance.

## 5. Summary and Conclusions

In this paper, we propose the TDGL, a fast multi-scale vision sensor module based on deep learning and designed for accurate and efficient road object detection. Instead of deepening the backbone network, we introduce the Transformation Dilated Grouped Layer feature extraction module, which adopts a custom lightweight three-branch structure. This design utilizes three-dimensional expansion rates at the end of each branch to effectively capture both detailed and global information. This normalized convolution unit enhances a model’s robustness. This multi-scale feature extraction method is particularly well suited to the complexities of autonomous driving environments, which often feature intricate backgrounds and targets of varying scales. By incorporating the TDGL module into the lightweight YOLO family model, we significantly enhanced the performance of this detection model on the BDD100K dataset. The proposed TDGL-based model surpasses leading two-stage detectors in accuracy while maintaining the high-speed inference advantage of lightweight architectures, making it an ideal vision sensor for real-time autonomous driving applications. 

## Figures and Tables

**Table 1:** Hyperparameter settings.

Hyperparameter	Values
epochs	100
optimizer	Auto
optimizer weight decay	0.0005
initial learning rate	0.01
final learning rate	0.01
imgsz	640
box loss gain	7.5
cls loss gain	0.5
dfl loss gain	1.5
warmup-epochs	3.0
warmup-momentum	0.8
warmup-bias-lr	0.10
mosaic augmentation	10
image HSV—saturation augmentation	0.7
image HSV—value augmentation	0.4
image HSV—hue augmentation	0.015
number of images per batch	16

**Table 2:** Comparison of improved results for multi-class models.

Methods	Conv	#Param	FLOPs	Precision/%	Recall/%	mAP@0.5/%	FPS
YOLOv3-tiny	—	12.1M	18.9G	38.7	21.3	19.7	61
YOLOv3-TDGL	GLConv	9.7M	18.2G	44.2	21.9	21.0	55
YOLOv5	—	2.5M	7.1G	36.6	27.3	25.7	50
YOLOv5-TDGL	GLConv	2.6M	7.2G	42.3	27.6	26.6	43
YOLOv6	—	4.2M	11.8G	39.7	26.2	25.1	64
YOLOv6-TDGL	Conv + BN	4.3M	11.9G	38.6	25.0	24.8	54
YOLOv6-TDGL	GLConv	4.4M	11.9G	39.3	26.3	26.6	53
YOLOv8	—	3.0M	8.1G	55.5	35.5	39.0	65
Faster-RCNN [4]	—	37.3M	92.8G	26.4	33.4	26.8	27
YOLOv8-TDGL	Conv + BN	3.0M	8.1G	55.1	34.3	38.5	57
YOLOv8-TDGL	GLConv	3.1M	8.2G	55.9	37.3	40.3	58

**Table 3:** Comparative experiments with SOTA object detection methods.

Methods	Backbone	#Param	FLOPs	Precision/%	Recall/%	mAP@0.5/%	FPS
ATSS [23]	ResNet-50	30.7M	92.1G	24.6	35.4	22.9	34
AutoAssign [24]	ResNet-50	31.4M	95.2G	28.3	34.8	27.0	33
YOLOv3-tiny	Tiny-Darknet	12.1M	18.9G	38.7	21.3	19.7	61
Faster-RCNN [4]	ResNet-50	37.3M	92.8G	26.4	33.4	26.8	27
CenterNet [25]	ResNet-50	25.8M	45.6G	29.9	37.4	29.0	32
Localization Distillation [26]	ResNet-18	10.1M	18.2G	22.6	30.4	20.7	38
YOLOv5	CSPDarknet	2.5M	7.1G	36.6	27.3	25.7	50
SSD300 [27]	VGG	21.6M	31.2G	24.3	31.9	18.6	45
FCOS [28]	ResNet-50	27.2M	38.9G	27.1	39.7	25.6	36
Generalized Focal Loss	ResNet-50	23.8M	25.8G	27.0	34.3	25.3	29
RetinaNet	ResNet-18	12.2M	16.8G	24.6	33.7	22.7	40
Ours	CSPDarknet	3.1M	8.2G	55.9	37.3	40.3	58

**Table 4:** Ablation experiment.

Groups	Expansion Rate	Relu	Softmax	L_relu	Depth	Channel	Precision/%	Recall/%	mAP@0.95/%	mAP@0.5/%
1	—	—	—	—	3\3\4	4	55.5	35.5	19.3	39.0
2	1\4\6	—	√	—	3\3\4	4	51.1	38.1	19.4	39.8
3	1\4\6	—	—	√	3\3\4	4	57.3	36.3	19.6	39.9
4	1\4\6	√	—	—	3\3\4	4	51.7	37.6	19.9	39.4
5	1\2\4	√	—	—	3\3\4	4	55.7	35.5	19.1	38.9
6	1\2\4	—	√	—	3\3\4	4	56.3	36.7	19.3	39.4
7	1\2\4	—	—	√	3\3\4	4	56.2	37.5	19.3	39.8
8	1\3\5	√	—	—	3\3\4	4	53.7	37.2	19.2	39.3
9	1\3\5	—	√	—	3\3\4	4	54.2	37.2	19.0	39.1
10	1\4\6	—	√	—	4\4\5	8	54.9	35.3	18.7	38.2
11	1\2\4	—	—	√	4\4\5	8	53.3	34.4	18.2	37.3
12	1\2\4	—	—	√	4\4\5	16	54.9	35.3	18.9	39.4
13	1\3\5	—	√	—	3\3\4	16	50.5	31.3	17.9	35.8
14	1\3\5	√	—	—	4\4\5	8	56.2	37.1	19.2	40.0
15	1\3\5	—	—	√	3\3\4	4	55.9	37.3	19.7	40.3

## Data Availability

The data presented in this study are available upon reasonable request from the corresponding author.
